# Draft Genome Sequences of Vibrio alginolyticus Strain S6-61 and Vibrio diabolicus Strain S7-71, Isolated from Corals in the Andaman Sea

**DOI:** 10.1128/MRA.01465-19

**Published:** 2020-02-20

**Authors:** Sushanta Deb, Jhasketan Badhai, Subrata K. Das

**Affiliations:** a Department of Biotechnology, Institute of Life Sciences, Bhubaneswar, India; University of Maryland School of Medicine

## Abstract

We report the draft genome sequences of Vibrio alginolyticus strain S6-61 and Vibrio diabolicus strain S7-71, isolated from the corals Pocillopora verrucosa and Fungia danai, respectively. The genomes of strains S6-61 and S7-71 contain 4,880 and 4,641 protein coding genes, respectively, and harbor genes associated with the ectoine biosynthesis pathway.

## ANNOUNCEMENT

Vibrio alginolyticus is a halo-tolerant mesophilic Gram-negative bacterium and has been characterized as an opportunist pathogen in humans and marine animals (1). Earlier studies have reported that the type III secretion system (T3SS) in Vibrio alginolyticus leads to severe fish disease, resulting in economic losses in the aquaculture industry (2). In contrast, Vibrio diabolicus is a heterotrophic, facultatively anaerobic, mesophilic bacterium, first isolated from an annelid Alvinella pompejana collected from a deep-sea hydrothermal vent (3). This bacterium can produce exopolysaccharide (EPS), which has importance in the biotechnological industry and human health (4). The identified ectoine and 2C-methyl-d-erythritol 4-phosphate (MEP) pathways in these bacteria are known to be associated with osmotic regulation and pathogenicity of bacterial cells (5, 6).

The bacterial strains used in this study were isolated from corals in the Andaman Sea. Coral samples were collected from Pocillopora verrucosa near North Bay (11°42′14.0″N, 92°45′05.7″E) and from Fungia danai near John Lawrence Island (12°01′33.8″N, 93°00′36.7″E). The isolation of bacteria and growth conditions were described earlier (7). Genomic DNA was isolated using the QIAamp DNA minikit (Qiagen, Germany). The quality (*A*_260/280_ ratio) and concentration of the DNA were determined using the NanoDrop 8000 UV-visible (UV-Vis) spectrophotometer and the Qubit 2.0 fluorometer (Thermo Fisher Scientific, USA). The DNA was sheared to an average length of 10 kb using a g-TUBE device, as per the manufacturer’s protocol (Covaris, Woburn, MA, USA). The fragmented DNA was used for SMRTbell library preparation as recommended by the manufacturer. The quantity and quality of the SMRTbell libraries were evaluated using the high-sensitivity double-stranded DNA (dsDNA) kit and Qubit fluorometer and the DNA 12000 kit on the 2100 Bioanalyzer (Agilent, Santa Clara, CA, USA), respectively. Sequencing was performed on the PacBio Sequel sequencing system (Pacific Biosciences, USA).

Quality control of the sequence reads was performed using the –correct and –trim parameters built into the Canu 1.3 program. *De novo* genome assembly of PacBio reads was performed with the Canu 1.3 assembler (https://github.com/marbl/canu/) (parameters: correct; p, bacteria; merylMemory, 15; batThreads, 12; stopOnLowCoverage, 100; genomeSize, 5.2m) (8). Scaffolding was performed using the Single Molecular Integrative Scaffolding (SMIS) pipeline (https://github.com/fg6/smis) (parameters: score, 50; len, 2000; step, 200; contig, 3000; edge, 5) (9). Finally, the gaps were filled with the help of PBJelly (parameters: minMatch, 8; minPctIdentity, 70; bestn, 1; nCandidates, 10; maxScore, 500; nproc, 8; noSplitSubreads) (10). A total of 680,654 and 1,057,603 PacBio reads were assembled into two draft genomes with sequencing coverage of ∼500-fold. A Perl script (https://github.com/tomdeman-bio/Sequence-scripts/blob/master/calc_N50_GC_genomesize.pl) was used to calculate the statistical elements of the assembled genome (Table 1). The draft genomes were annotated using the NCBI Prokaryotic Genome Annotation Pipeline (PGAP version 4.9) with default parameters (11). The final draft genome assemblies of strains S6-61 and S7-71 are summarized in Table 1. Putative pathways in the bacterial genomes were identified using the KEGG pathway analysis tool (12). The Clusters of Orthologous Groups (COG) functional categories of the predicted protein coding genes were identified using the Perl script cdd2cog (https://github.com/aleimba/bac-genomics-scripts/tree/master/cdd2cog) (13).

**TABLE 1:** Characteristics of the draft genome sequences and accession numbers of the two *Vibrio* strains

Strain	Genome size (Mbp)	GC mol%	No. of scaffolds	No. of reads	Avg read length (bp)	*N*_50_ (Mbp)	No. of CDS^*a*^	% genes coding proteins	No. of tRNAs	No. of rRNAs
S6-61	5.3	44.52	9	680,654	2,600	1.9	5,035	96.9	92	21
S7-71	5.1	44.89	5	1,057,603	2,706	1.8	4,816	96.3	133	44

a CDS, coding DNA sequences.

Comparative genomic analysis was performed for identification of the strains described in this study. Strains S6-61 and S7-71 showed 99.87% and 99.68% 16S rRNA gene sequence similarity to Vibrio alginolyticus strain NBRC 15630 and Vibrio diabolicus strain LMG 3418, respectively. In addition, the average nucleotide identity (ANI) was determined using the JSpeciesWS server (14). The ANI relatedness of strain S6-61 with the reference strain Vibrio alginolyticus NBRC15630 was 98.12%. Similarly, strain S7-71 had an ANI relatedness of 97.94% with the reference strain Vibrio diabolicus LMG 3418. These values are above the threshold ANI value (96%) for species delineation (15), suggesting that strains S6-61 and S7-71 belong to the species Vibrio alginolyticus and Vibrio diabolicus, respectively. Furthermore, *in silico* DNA-DNA hybridization (isDDH) values between strain S6-61 and Vibrio alginolyticus strain NBRC 15630 and between strain S7-71 and Vibrio diabolicus strain LMG 3418 were 85.60% and 83.30%, respectively, which are above the well-recognized cutoffs (≥70% isDDH) for bacterial species delineation. COG functional analysis revealed that the respective genomes of strains S6-61 and S7-71 contain genes involved in carbohydrate transport and metabolism (4.4% and 4.8%), lipid transport and metabolism (2.6% and 2.9%), transcription (7.1% and 6.8%), signal transduction mechanisms (5.6% and 6.0%), and unclassified functions (13.9% and 11.11%).

The presence of predicted genes for ectoine biosynthesis suggests that these bacteria can resist osmotic stress in marine environments. In addition, the MEP pathway in V. alginolyticus strain S6-61 can be used as a potential drug target.

### Data availability.

The whole-genome shotgun sequences of strains S6-61 and S7-71 have been deposited in DDBJ/ENA/GenBank under the accession numbers WAHT00000000 and VYYA00000000, respectively (Table 1). The SRA data are available at the NCBI SRA database under the accession numbers SRR10194733 and SRR10194627, respectively.
